# Supplementary material for: Polyketide synthase gene clusters in the ericoid mycorrhizal fungus Oidiodendron maius: genome-wide prediction, expression profiling, and functional characterization of knockout mutants
Source: Front Plant Sci. 2026 Sep 16;17:1924430. doi: 10.3389/fpls.2026.1924430 (PMC13623903; doi:10.3389/fpls.2026.1924430)
Supplement: Supplementary file 1 [file SupplementaryFile1.zip › Supplementary Materials/Supplementary Folder 1.zip!/Oidma1_AssemblyScaffolds_Repeatmasked/knownclusterblast/cluster49/jgi.p_Oidma1_136488_mibig_hits.html]

| MIBiG Protein | Description | MIBiG Cluster | MiBiG Product | % ID | % Coverage | BLAST Score | E-value |
| --- | --- | --- | --- | --- | --- | --- | --- |
| EHA28243.1 | hypothetical\_protein | BGC0001143 | Azanigerone\_biosynthetic\_gene\_cluster | 33 | 93.1863727455 | 214 | 2.5e-55 |
| ADY16695.1 | TqaG | BGC0001142 | Tryptoquialanine\_biosynthetic\_gene\_cluster | 28 | 95.5911823647 | 201 | 2.8e-51 |
| EAL89048.1 | FAD\_binding\_domain\_protein | BGC0000355 | Fumiquinazolines\_biosynthetic\_gene\_cluster | 30 | 96.1923847695 | 190 | 5e-48 |
| EAA65603.1 | hypothetical\_protein | BGC0000022 | Asperfuranone\_biosynthetic\_gene\_cluster | 29 | 100.601202405 | 183 | 7.9e-46 |
| BAD83683.1 | FAD/FMN-dependent\_oxygenase/oxidase | BGC0000012 | Alternapyrone\_biosynthetic\_gene\_cluster | 29 | 97.3947895792 | 173 | 6.3e-43 |
| EHA28234.1 | FAD/FMN-containing\_dehydrogenase | BGC0001143 | Azanigerone\_biosynthetic\_gene\_cluster | 28 | 90.9819639279 | 164 | 2.9e-40 |
| EAL92288.1 | FAD\_binding\_domain\_protein | BGC0000372 | Hexadehydro-astechrome\_(HAS)\_biosynthetic\_gene\_cluster | 26 | 87.9759519038 | 113 | 7.7e-25 |
| AAM97372.1 | RubI | BGC0000266 | Rubromycin\_biosynthetic\_gene\_cluster | 32 | 47.6953907816 | 102 | 1.8e-21 |
| ANY57959.1 | MtcA\_FAD-linked\_oxidase | BGC0001369 | Metatricycloene\_biosynthetic\_gene\_cluster | 30 | 44.0881763527 | 98 | 2e-20 |
| ABI22120.1 | oxidoreductase | BGC0000422 | Saframycin\_A\_biosynthetic\_gene\_cluster | 32 | 39.6793587174 | 93 | 6.3e-19 |
| AAD28454.1 | MitR | BGC0000915 | Mitomycin\_biosynthetic\_gene\_cluster | 31 | 39.6793587174 | 91 | 2.4e-18 |
| ABP55175.1 | FAD\_linked\_oxidase\_domain\_protein | BGC0000150 | Sporolide\_biosynthetic\_gene\_cluster | 24 | 89.5791583166 | 89 | 9.1e-18 |
| CDM31316.1 | FAD-binding,\_type\_2 | BGC0000667 | PR\_toxin\_biosynthetic\_gene\_cluster | 32 | 32.6653306613 | 79 | 1.6e-14 |
| EAQ86396.1 | hypothetical\_protein | BGC0001405 | Chaetoviridin\_/\_chaetomugilin\_biosynthetic\_gene\_cluster | 23 | 85.7715430862 | 65 | 2.4e-10 |
| ADY76663.1 | FAD-depending\_oxidoreductase | BGC0000950 | Napsamycin\_biosynthetic\_gene\_cluster | 31 | 37.2745490982 | 61 | 2.7e-09 |
| ADN26247.1 | FAD-dependent\_oxidoreductase | BGC0000951 | Pacidamycin\_biosynthetic\_gene\_cluster | 27 | 39.4789579158 | 60 | 5.9e-09 |
| ACZ66255.1 | APS9 | BGC0000304 | Apicidin\_biosynthetic\_gene\_cluster | 27 | 35.4709418838 | 57 | 3.8e-08 |
